# Observing Picomolar Protein Unfolding Using Resonance Light Scattering

**DOI:** 10.3390/biom15111579

**Published:** 2025-11-10

**Authors:** Alain Bolaño Alvarez, Kristian Bakke Arvesen, Kasper Fjellhaugen Hjuler, Peter Bjerring, Steffen B. Petersen

**Affiliations:** Department of Dermatology and Venerology, Aalborg University Hospital, Hobrovej 18-20, 9000 Aalborg, Denmark; kristian.bakke.arvesen@gmail.com (K.B.A.); kf.hjuler@rn.dk (K.F.H.); email@peterbjerring.dk (P.B.)

**Keywords:** melting point, resonance light scattering, picomolar concentration, BSA, botulinum toxin A, protein stability, melting transitions

## Abstract

We here present a novel and sensitive methodology for determining the melting point (MP) of Bovine Serum Albumin (BSA) from micromolar to picomolar concentration levels under label-free conditions. At 1 pM we could model the melting with a sharp Gaussian. However, from the transient state observed during the melting process by using a simple exponential decay model, we determined a time constant of 67 s. We applied this methodology by studying a 3.3 pM sample of a botulinum toxin A (BoNT-A) (stabilized with 2.8 nanomolar denatured Human Serum Albumin (HSA)). We were able to determine the Tm of BoNT-A in the presence of approximately 1000-fold more concentrated HSA. This method enables the detection of protein melting transitions at picomolar concentrations without the use of a fluorescence dye. Its sensitivity and simplicity make it a valuable analytical tool for studying protein stability in diluted pharmaceutical formulations. This method is useful for correlating thermal conformational changes with catalytic function.

## 1. Introduction

The detection of protein unfolding at ultralow concentrations—defined here as the pico- to nanomolar range—is crucial in biopharmaceutical studies, where protein stability in biopharmaceuticals such as botulinum neurotoxins is essential, as they function at extremely low concentrations but must retain their 3D conformation to translocate to the neuronal cytosol [1]. At these concentrations, intermolecular aggregation is minimized, allowing intrinsic conformational transitions to be examined under near-native conditions [2,3]. Conventional techniques, including circular dichroism, infrared spectroscopy, and differential scanning calorimetry, typically require micromolar protein concentration [4]. Thus, aggregation often masks the native melting transition.

Fluorescent dye-based methods, such as thermal shift assay, improve sensitivity and enhance the signal-to-noise ratio to achieve detection in the millimolar (mM) range [5], and nanomolar range in the case of the Bradford method [6]. However, the dye used can potentially modulate both the transition and stability of the protein as well [7]. While dye-binding, such as Sypro or ANS, achieves high sensitivity, their utility at picomolar concentrations for detecting Tm is limited due to the inability to isolate conformational changes from dye-induced effects [5,7].

Label-free techniques, such as Circular Dichroism (CD) and Dynamic Light Scattering (DLS), have been widely applied to study protein unfolding and aggregation [8,9,10]. However, these methods generally require protein concentrations in the micromolar-to-millimolar range to achieve measurable signals, making them unsuitable for detecting thermal transitions at ultralow (picomolar) concentrations [9,10,11]. Furthermore, while CD provides averaged secondary structure information [9], and DLS reports on particle size and aggregate formation [11], none of these techniques can capture the intrinsic conformational dynamics (time constant) of ultralow-concentration protein in solution. Therefore, a clear need remains for a simple, dye-free, and highly sensitive optical method for tracking protein folding and stability under near-physiological and dilute conditions.

Here, we are dealing with a special case of scattering that is called Resonance Light Scattering (RLS) to study protein folding dye-free, exploiting the intrinsic chromophores (Trp, Tyr) whose resonance scattering signal changes with the protein conformation. This phenomenon is observed if the excitation wavelength used is the same or near the wavelength of absorption of the chromophore, and particularly, the magnitude of the effect increases in aggregate states [12] which can enhance the observed change in response to the protein melting process.

This RLS approach was validated by employing a pharmaceutical product, BOCOUTURE, which has HSA as a stabilizer molecule and BoNT-A as the active principle. The stabilizers can be molecules of low molecular weight, such as caprylate and acetyltryptophanate, in the case of commercially available HSA [13] or a highly stable protein with a well-defined structure, such as HSA [14,15] which has been used to stabilize proteins in lyophilized formulations [16] such as BOCOUTURE. The mechanism of action of these stabilizer molecules follows different rules: they interact directly with the active substance (caprylate and acetyltryptophanate) [13] or the active principle can be shielded to avoid self-aggregation, as HSA does with BoNT-A [17]. In this case, its concentration is higher than the active principle.

The stabilizing role of HSA is based on its high stability. Thus, HSA can maintain the structural integrity of the active principles (BoNT-A), acting as a scaffold to prevent conformational changes [18,19]. In addition, it reduces the likelihood of aggregation triggered by self-interactions [17] and stabilizes the pH within the formulation [20,21].

BoNT-A is clinically utilized for diverse conditions, including chronic migraine, cervical dystonia, muscle spasticity, strabismus, overactive bladder, and hyperhidrosis [22]. In esthetic dermatology, it is extensively employed for reducing facial wrinkles, such as frown lines, crow’s feet, and forehead lines, as well as for softening neck wrinkles and marionette lines [23,24]. Thus, formulation improvements are a central issue in the pharmaceutical industry aimed at clinical applications. Two crucial points are emphasized in the formulation field: (1) the stabilizer role in preserving the activity of the active substance, and (2) the sensitivity of the methodology employed to conduct stability and quality control studies.

**Figure 1:**
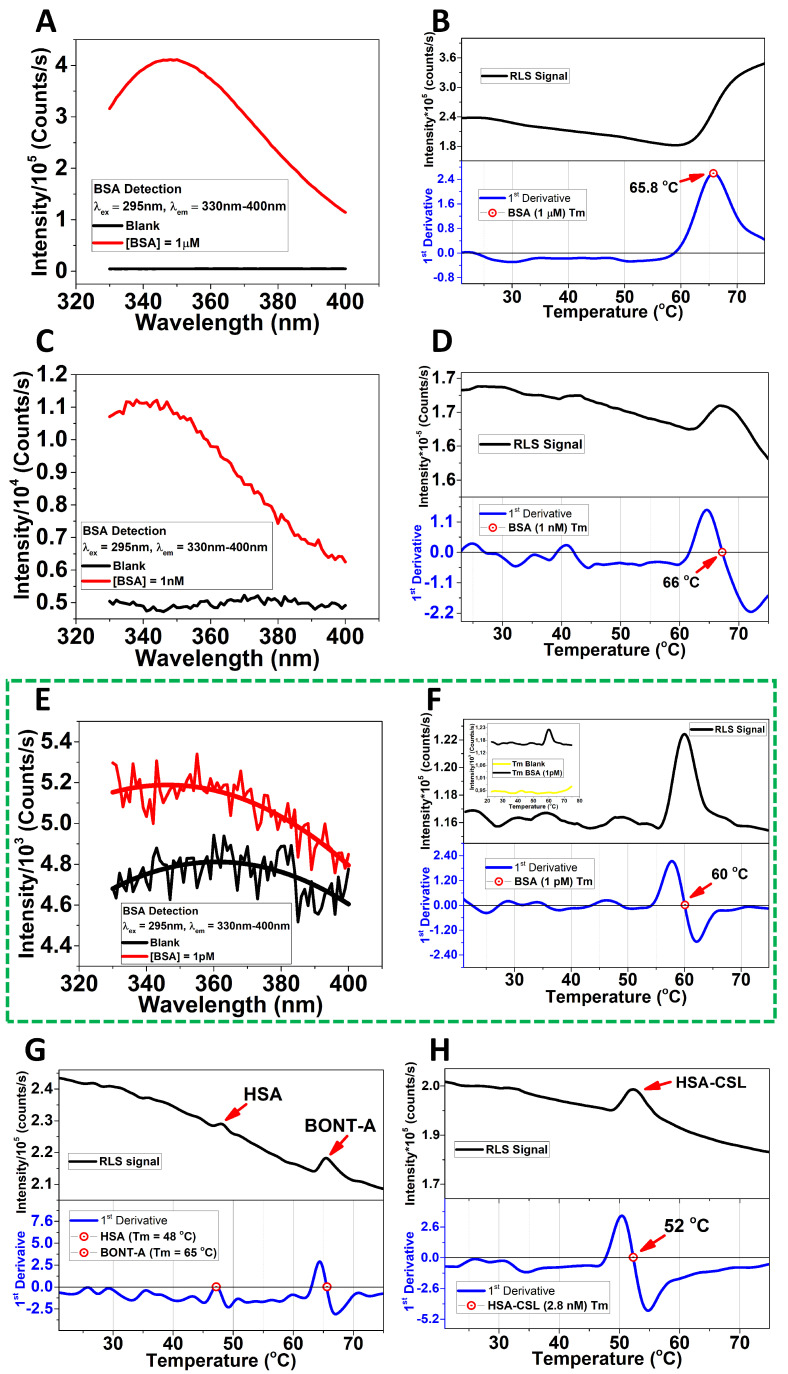
Melting point determination and detection of BSA at ultralow concentration without fluorescent dyes. No evidence of aggregation was detected at 1 pM for the BSA sample. BSA detection (**A**) and MP (**B**) at 1 µM; BSA detection (**C**) and MP (**D**) at 1 nM (69 ± 3 °C); and BSA detection (**E**) and MP (**F**) at 1 pM concentrations. The insert remarks the difference between blank and 1 pM BSA concentration. The excitation wavelength was at 295 nm, and the emission range was between 330 nm and 400 nm. The average melting point curve corresponding to BSA at 1 pM (59.09 ± 1.28 °C, After Correction: 61.79 °C); the insert shows the signal with respect to the blank (**F**). Melting point of the BoNT−A (65 °C) at 3.3 pM and HSA (48 °C) at 2.8 nM in BOCOUTURE (**G**). The melting point of the HSA (52 °C) at 2.8 nM of concentration determined by the standard calibration curve by using a commercial HSA−CSL sample (**H**). The thermal scans were in the optimal optical properties of the cuvettes. See SI for scans in the range 20−90 °C. The raw data were processed in Origin Pro.

In the present study, we have established unequivocal evidence that RLS can be used to monitor the ‘melting’ of selected proteins (BSA, HSA, and BoNT-A) down to micromolar concentrations using a dye-free approach. The methodology monitors the experimental melting temperature as a function of protein concentrations from 1µM to 1pM, under thermal scan range from 20 °C to 75 °C and/or 20 °C to 90 °C (Figure 1 and Figure S1).

## 2. Materials and Methods

Materials: Bovine serum albumin (BSA) (Lot: SLBM5216V) was acquired from Sigma-Aldrich (St. Louis, MO, USA) and was dissolved in either phosphate-buffered saline (PBS) at pH7 from VWR, BDH Chemicals (Hackettstown, NJ, USA) (Lot: 23F214144) or water, Milli Q Type II. Quartz cuvettes were acquired from Hellma Analytics from Müllheim, Germany (required an extreme cleaning process to work at ultralow protein concentrations), and acrylic cuvettes were from Sarstedt (Germany). Human Serum Albumin (HSA) (Lot: P100491951) was acquired from CSL Behring GmbH, Tyskland, Germany, and Dry Botulinum Toxin formulation (BOCOUTURE) (Lot: 139059) was from Merz Pharmaceuticals GmbH, 60318 Frankfurt/Main, Tyskland, Germany; each preparation was dissolved in NaCl 0.9% (Lot: 22452413) from B. Braun Melsungen AG, 34209 Melsungen, Germany.

Fluorescence spectra: Fluorescence spectra were recorded using a Horiba Scientific Spectrofluorometer Model with a 75 W Xenon ARC lamp (Ushio Inc., Tokyo, Japan) in a cuvette holder with a temperature control system at 25 °C, and a 10 × 2 mm quartz cell designed for a low volume. The excitation and emission wavelengths were 295 nm and 350 nm, respectively, with excitation slits of 1.4 mm and 10 mm, and emission slits of 20 mm and 30 mm, to improve the light input in the PMT (Photomultiplier Tube). The integration time was 10 s, and the scan rate was 1 nm/s.

Determination of the HSA (stabilizer) concentration in the BOCOUTURE sample and preparation of BoNT-A from dry formulation: Dry botulinum toxin from Merz Pharmaceuticals GmbH (BOCOUTURE^®^) (Frankfurt/Main, Tyskland, Germany) was prepared in NaCl 0.9%. According to the International Unit System, 20 IU = 1 ng. One vial containing 50 IU (2.5 ng) of BoNT-A was dissolved in 0.005 L of 0.9% NaCl to obtain 3.33 pM of BoNT-A. To determine the concentration of Human Serum Albumin (HSA) in the BOCOUTURE sample, an HSA-CLS calibration curve was employed. The BOCOUTURE sample contained both HSA (unknown concentration) and BoNT-A at 3.3 pM, allowing the HSA stabilizer concentration to be calculated by subtracting the BoNT-A concentration (3.33 pM) from the total protein concentration.

Measurement of total protein concentration in the BOCOUTURE sample: A sample of the BOCOUTURE dry formulation, containing both HSA (unknown concentration) and BoNT-A at 3.3 pM, was subjected to absorbance measurements at 280 nm in a UV-Vis spectrophotometer DS5. The measured absorbance corresponded to the total protein concentration of the sample, which included both HSA and BoNT-A.

Preparation of HSA-CLS calibration curve and calculation of HSA stabilizer concentration in BOCOUTURE: Pure HSA-CLS was used to create a calibration curve (R^2^ = 0.99) for measuring absorbance at 280 nm in a UV-Vis spectrophotometer DS5. Different dilutions of pure HSA-CLS were prepared using a NaCl 0.9% solution. These dilutions were prepared meticulously to ensure accurate measurements. The curve allowed the calculation of HSA stabilizer concentration [HSA] = [total protein]-[BONT-A] = 2.8 nM−3.3 pM ≈ 2.8 nM in the BOCOUTURE, which is essential for accurately determining the melting point of the HSA stabilizer.

Absorbance measurements: Absorbance measurements of the HSA dilutions were carried out at 280 nm using a precision absorbance instrument, the DS5 UV-VIS spectrophotometer from Edinburgh Instruments Ltd., Livingston, UK. The instrument was calibrated according to the manufacturer’s specifications. In addition, before the measurements were taken, a baseline was established.

**Figure 2:**
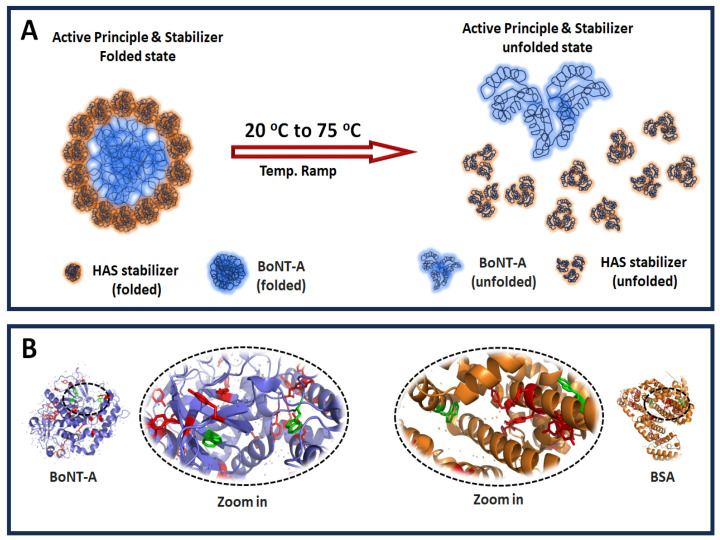
Temperature effect on the protein conformation and relative chromophores proximity. (**A**) Schematic representation of the temperature effect on the active principle and stabilizer folded structure. (**B**) Relative proximity between tyrosine (red) and tryptophan (green) amino acids. The average distance between Trp residues is 49.35 Å (BSA, PDB: 4f5s). PyMol (Version 3.1) was used for visualization.

Calculation of HSA-CLS concentration: The absorbance values obtained from the HSA-CLS dilutions were used to generate a standard calibration curve by plotting the absorbance versus known HSA-CLS concentrations. This curve served as a reference to determine the HSA stabilizer concentration in the BOCOUTURE sample (see Figure 2).

This simple method allowed for accurate quantification of HSA stabilizer in the BOCOUTURE sample, facilitating the subsequent analysis of protein stability and melting point determination in the context of this pharmaceutical product.

Melting point determination: The melting points of both BSA and BoNT-A were recorded using the same instrument to obtain the fluorescence spectra using a scattering setting coupled to a ramp temperature (rate = 2 °C/min). The temperature varied from 20 °C to 75/80 °C in pure milli Q water and PBS pH7 (the results did not change significantly). The excitation emission wavelength was 295 nm. The S/N of the spectra was improved by using 0.1 points/sec in the recording rate. The accuracy of the melting point is shown by the first derivative.

Removing bubbles in the sample: The reduction in the bubble content in the sample was enhanced through the following methods:

1. The vacuum degassing process which minimized potential sources of interference in the scattering analysis.

2. Heating process in the same temperature range. Increasing the temperature reduced the solubility of the gases in the solution, facilitating the escape of bubbles from the solution to the air [25].

**Figure 3:**
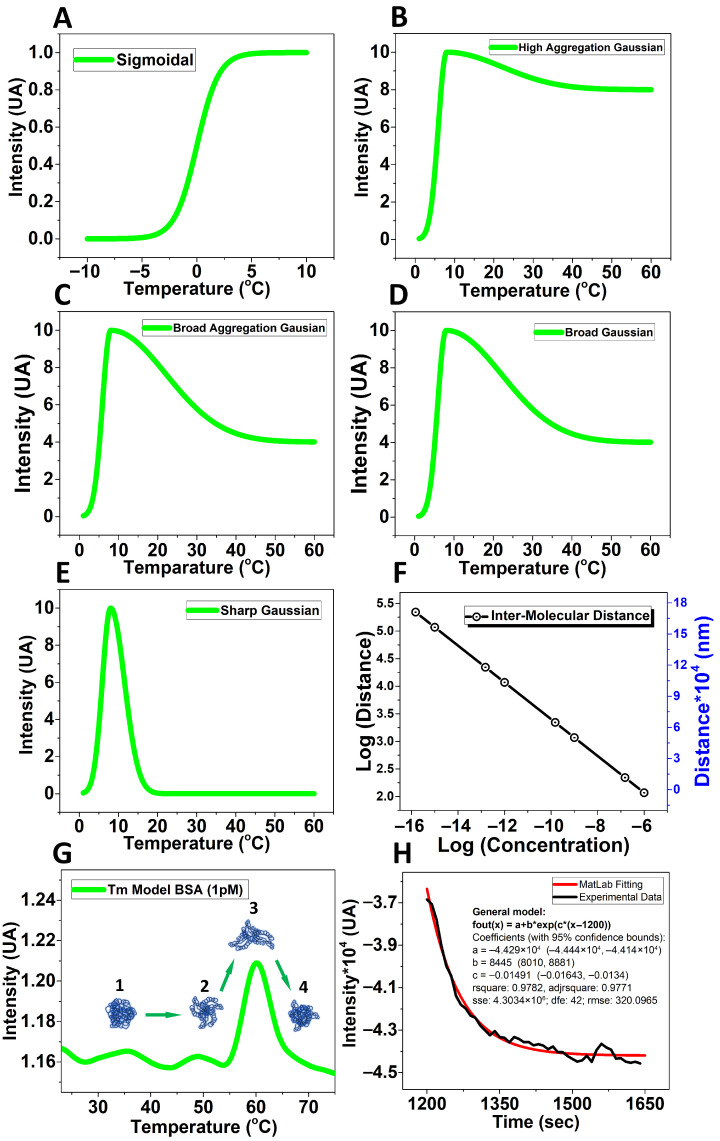
Mathematical model from MATLAB. The curves from (**A**–**E**) use dimensionless coordinates to illustrate the qualitative evolution of the unfolding/refolding or aggregation transitions observed experimentally (see Supporting Information). The axes represent relative temperature and relative intensity rather than absolute experimental units. (**A**) The mathematical model contemplates the concentration values fleeting from a Sigmoidal; (**B**) High aggregation Gaussian; (**C**) Broad aggregation Gaussian; (**D**) Broad Gaussian; (**E**) Sharp Gaussian; (**F**) Molecular distance change with respect to the concentration; (**G**) Proposed model to explain the experimentally observed behavior at 1 pM with: (**1**) native BSA structure, (**2**) before unfolded BSA structures, (**3**) unfolded BSA structures, and (**4**) refolded BSA structure (new globular structure); (**H**) Exponential decay kinetic describing the collapsing protein structure into the new globular conformation after unfolding at Tm (see General model parameters in the insert).

MATLAB model: The model (Figure 3A–E) represents a dimensionless simulation of the unfolding/refolding and aggregation processes contributing to the RLS signal. The X-axis corresponds to a relative temperature coordinate, and the Y-axis to relative scattering intensity. These functions qualitatively reproduce the transition shapes observed experimentally (see Figure S1) rather than reporting absolute measured values.

Single exponential decay to evaluate the time constant: Passing the melting point, the behavior following a single exponential decay kinetic (*τ*_1pM_ = 67.07 s and *τ*_1nM_ = 529 s) according to MATLAB calculations (version R2023a), the general model for 1pM is giving by the next equation: f(x) = a + b*exp(c*(x − 1200), where a = −4.429 × 10^4^, b = 8445, c = −0.01491 with a 95% of confidence and r^2^ = 0.9771 (Figure 3H), 1200 denotes the location of the peak of the thermal transition.

## 3. Results

In structural studies of proteins, detection at ultralow concentrations is challenging. Our results indicate effective detection, showing a clear tendency toward a decreasing signal-to-noise ratio as the BSA concentration decreases, with the ratio remaining greater than unity [3] (Table 1).

When a folded protein is experiencing progressive heating from close to 0 °C to nearly 100 °C, it most likely will ‘melt’ at some intermediate temperature. This melting can be perceived as a phase transition; the protein’s folding state changes from the initial state to a new state within a few degrees Celsius. Most often, this is an irreversible process; cooling does not restore the original low-temperature folded state. Somehow, the protein becomes stuck in new constraints that cannot be overcome in the unfolded state, where it is now populated. Various spectroscopic methods can monitor this process: circular dichroism, fluorescence spectroscopy, infrared spectroscopy, and light scattering. Traditionally, the determination of the melting point of proteins such as BSA has been confined to relatively high concentrations, where the cooperative unfolding transition is readily detectable [2], which was an effect we observed clearly at 1 µM (Figure 1B). Here, we determined the melting point of BSA at different concentration from 1 pM up to 1 µM by RLS, exciting at 295 nm and scanning from 20 to 75 °C and/or 20–90 °C (see Supporting Information), the Tm was corrected by plotting the data from Table 2: Correction = (Tm*1.05) − 0.25, where Tm is the experimental melting point in °C, 1.05 is the slope of the plotted data, and −0.25 is the intercept of the plotted data. This correction improves the accuracy of the melting point values, reducing the error introduced by the instrument temperature control system in the holder. The accuracy of the set temperature in the holder was checked using an electronic temperature tester.

Certainly, at 1 pM the self-aggregation is likely to be less pronounced (Figure 1F). However, most proteins contain multiple aromatic residues that sense the excitation state of the surrounding aromatic residues [26] which we propose is relevant for detecting the MP at ultralow concentration. We may enhance the scattering intensity by up to 10-fold because many tryptophan and tyrosine residues can contribute to the observed resonance scattering effect by mimicking a self-aggregation state due to their proximity in the same protein structure (Figure 2). Our methodology provides a visual representation of the unfolding/refolding transitions of BSA molecules, proposing a model to explain this behavior at ultralow concentrations (Figure 3E,G,H). We can support our experimental findings by comparing the experimentally determined melting points with predictions obtained using mathematical tools (Figure 3).

To explain the proposed model, our data show distinctive changes in the RLS profile when the temperature increases. These alterations in the light scattering intensity correspond to the structural transitions within the BSA molecules, determining different levels of self-exposure of Trp, especially at the temperature range over which the melting occurs, which is centered around 60 °C (Figure 1F). Interestingly, we are also able to evaluate the time needed for the transient unfolding event (*τ*_1pM_ = 67.07 s and *τ*_1nM_ = 529 s), since we can measure the duration of the enhanced resonance scattering signal by using a single exponential decay (see Supporting Information). We have measured this at all concentrations, and the result is shown in Figure S1.

According to our understanding, no other label-free method for studying protein melting has been reported for concentrations as low as picomolar.

Currently, this method is therefore unique, and we postulate that it may be the only known method where the protein melting process can be studied without aggregational processes contributing to the thermodynamics of the melting process at extreme dilution. In other words, the apparent melting point is most likely shifted because of the aggregation stabilizing the unfolded state at high concentration, which relates to available studies [4,27]. We have monitored the resonance light scattering method at a range of concentrations. At low concentrations, the light scattering trace appears as a skewed Gaussian with a sharp onset and a slow trailing decay. We believe the sharp onset is caused by a sudden increase in the physical size of the protein because of a partial unfolding. We suggest the trailing decay is the melted protein seeking a new, possibly globular but misfolded conformation (Figure 1F and Figure 3G). When concentration increases, the trailing decay lasts longer, indicating a more complex search for a new conformation. Increasing the concentration further, we start observing that the protein retains its higher scattering response—indicating that its melted, deformed size does not relax back to the levels observed at lower protein concentration. However, the time constant cannot be determined at high concentration (1 µM) since the aggregation process dominates over refolding (Figure 1). Although other methodologies such as digital ELISA can achieve femtomolar sensitivity by detecting single protein molecules complexes on antibody-coated beads [28] it requires labeling and immobilization, whereas our RLS approach provides conformational information from unlabeled proteins directly in solution.

In this study, to demonstrate our dye-free melting point determination approach, we used a Botulinum Toxin (3.3 pM) formulation (BOCOUTURE), which contains HSA (2.8 nM) as a stabilizer. The HSA concentration in BOCOUTURE was determined by constructing a standard calibration curve employing a commercially available HSA-CLS (Behring GmbH) (Supporting Information). The analysis revealed two distinct melting points within the BOCOUTURE. The first was observed at 48 °C and the second at 65 °C (Figure 1G). To identify the melting point of HSA within the BOCOUTURE, a sample of HSA-CLS was subjected to RLS under the same experimental conditions. The result showed that the melting point of HSA-CLS was 52 °C (Figure 1H). We have corrected the melting point values by using our previous proposed model (Table 2 and Table 3).

In addition, we have not detected evidence of aggregation in the 1 pM BSA sample, as verified in a backscattering instrument. In contrast, a 100-fold diluted Ozempic API (semaglutide) sample was analyzed as an external positive control, which exhibited aggregates with a hydrodynamic diameter of approximately 107 nm.

## 4. Discussion

The change in the RLS signal to determine the melting points of the proteins matches the proposed mathematical model, supporting that the proteins undergo a globular conformational re-arrangement that differs from the native structure, which explains the recovered scattering intensity at 1 nM and 1 pM (Figure 1D,F–H). Our analysis at 1 pM did not include aggregation because the intermolecular distance at 1 pM (117,274.3 nm) makes aggregation extremely unlikely (Figure 3F). We assume that the unfolding is truly a mono-molecular process resulting in a transient change in the protein size, followed by a relaxation to a new folding state, even if the stabilizer (HSA) forms a shell to protect the active protein (BoNT-A) of self-aggregation, disassembling such scaffold by the temperature scan, thus detecting separately both melting points (Figure 1G and Figure 2A).

Following the melting transition of BSA, the RLS signal displayed a sharp onset followed by a decay, consistent with a first-order relaxation dominated by intramolecular refolding of a homogeneous BSA population at both 1 pM and 1 nM, rather than multimeric aggregation. Accordingly, the post-transition trace was successfully fitted using a single-exponential decay function (R^2^ = 0.9771), allowing us to calculate the time constant (τ_1pM_ = 67 s; τ_1nM_ = 529 s). Therefore, the addition of a second exponential term did not improve the fit, indicating the absence of kinetically distinct BSA subpopulations at both 1 pM and 1 nM.

This RLS method was improved by combining vacuum degassing and preheating of the buffer solution to eliminate bubbles and gas-related interferences, which significantly enhanced the signal quality in melting point determination.

These improvements make the methods valuable for routine spectroscopic applications to analyze proteins, while minimizing the required sample concentration.

An important factor to highlight is the sensitivity of the RLS method that primarily arises from the resonant excitation of aromatic amino acids, mainly tryptophan and tyrosine, whose absorption bands overlap with the excitation wavelength (295 nm), enhancing the scattering response [12]. In this case, it is important to consider the quantum yield (QY) of both residues, which changes with the environment, the solvent polarity [29] and decreases when the temperature increases between 25 °C and 95 °C [30], which is relevant in thermal scans for tracking both Trp and Tyr during conformational changes in the protein structure. In addition, at 295 nm, excitation is highly selective for tryptophan residues, whose indole side chains dominate protein intrinsic fluorescence [31]. It explains why the 295 nm over 280 nm enhances the RLS signals. However, proteins with a lower abundance of aromatic residues, such as HSA compared to BoNT-A, exhibit weaker responses (see Figure 1G), also influenced by the intrinsic QY and expositions of these residues during the thermal scan. Moreover, intrinsic chromophores, such as the heme group in hemoproteins, can act as alternative scattering centers, extending the applicability of RLS to proteins with absent aromatic residues while maintaining a label-free character.

We believe that our result is important for mainly two reasons: First, we are observing the unfolding event without any dye being introduced into the sample. Second, we can observe the label-free unfolding event at a concentration level not achieved by any other biophysical method. Interestingly, we are also able to evaluate the time needed for the transient unfolding event, since we can time the duration of the enhanced resonance scattering signal. This highlights how ultralow concentration affects the stability of proteins, such as BSA, HSA, and BONT-A. This methodology is innovative and useful to study biopharmaceutical formulations where BSA or HSA are used as a stabilizer [32], as well as formulations based on proteins that are extremely diluted, such as botulinum toxin [18]. However, if the stabilizer is a molecule with low molecular weight, such as tryptophanate (tryptophan anion) and caprylate (octanoic acid), as occurs in the case of HSA-CSL preparation [13,33], some differences in the melting point can emerge. This was the reason for the observed differences between the melting points of HSA-CLS (52 °C) and HSA stabilizer within the BOCOUTURE (48 °C) (Figure 1G,H and Table 1). However, the conformational behavior of both proteins (BONT-A and HSA) from the folded state to a refolded state remains differentiable and which are well detected by this methodology. Our study can help to understand how stabilizing molecules impact the melting point to trigger new protein conformational behavior (refolding) during thermal stability analysis.

Beyond BSA, HSA, and BoNT-A, the method was successfully extended to lipidated peptides such as semaglutide (Figure S3), demonstrating that RLS can resolve multiple thermodynamic transitions even in non-globular proteins.

Additionally, to assess the reproducibility and stability of the RLS method, BOCOUTURE samples were incubated at −20 °C, 4 °C, 20 °C, and 37 °C. Consistent and well-defined Tm values were observed at −20 °C and 20 °C (Figure S2 and Table S1), while samples stored at 4 °C and 37 °C showed weak or undetectable transitions. It indicates a kinetically trapped conformation (highly structured and hydrated native state) at 4 °C, while progressive destabilization and possible degradation occur at higher temperatures (37 °C). These findings align with storage guidelines from the Centers for Disease Control and Prevention (CDC) and U.S. Food and Drug Administration (FDA), which recommend refrigeration (2–8 °C) and single-use within 4 h after reconstitution due to potency loss and degradation risks [34,35]. Similar studies have shown that BoNT-A retains biological activity for up to 4 weeks below 4 °C but loses potency when this threshold is exceeded [36]. Together, these results highlight the robustness of the RLS method for tracking conformational changes in biopharmaceutical formulations under clinically relevant storage conditions. Thus, this study is particularly relevant for hospital and clinical applications, where BoNT-A formulations are reconstituted and used within 4 h after saline (NaCl 0.9%) reconstitution, following CDC recommendations and FDA guidelines, which specify single-use vials for each preparation [34,35]. However, several clinical practitioners have revealed that up to 67% of store and reuse reconstituted BoNT-A vials for 1–4 weeks, contrary to official recommendations, due to cost and maintenance of efficacy [35].

We suggest extending the RLS approach to monitor enzyme activity concurrently with melting transition scans, which offer a dynamic view of structural stability and catalytic function. This innovative application could uncover how thermal unfolding and refolding relate to enzymatic activity even in intermediate conformations, providing new insights into protein thermodynamics beyond traditional fluorescence.

Finally, an enhanced version of this RLS methodology could also be applied to investigate the kinetic heterogeneity recently reported by Jiang et al., in protein refolding states [37]. The ability of RLS to monitor conformational changes in real time provides a label-free tool to distinguish single- and multi-exponential relaxation processes and detect misfolded intermediate states.

### Signal Intensity Changes in the Sample Volume

Modern photon-sensing technology has progressed in the most impressive manner. Many cameras declare a quantum efficiency of 0.5 or better for some ranges of wavelengths [38]. When considering the full spectral width, the quantum efficiency may drop to 0.1 at the least sensitive wavelength [39]. In simple absorption terms, we can detect a few non-aggregated molecules. If we consider fluorescence, the exciting photons are covered as part of the fluorescence process by a longer wavelength photon. Most often, the wavelength shift is relatively small, thus the quantum efficiency is nearly unchanged. If we consider the folding/unfolding process of proteins, we will excite the molecule periodically and monitor the absorption or fluorescence as a function of time. Here, we expect a near-exponential growth or disappearance of a signal whose intensity is assumed to be indicative of the population of the molecule in a particular folding state. This change in signal intensity must stem from photon detection from a statistical subset of molecules (Figure 1 and Figure S1). If we assume the sample volume we can detect a signal from is 3000 uL, and the concentration of our molecule of interest is 1 pM, we are observing the signal from N = 1.8 × 10^9^ molecules. This novel approach avoids interferences introduced by fluorescent dye and offers a novel insight for ultrasensitive protein analysis in the pharmaceutical industry, especially at ultralow protein concentrations, highlighting that the globular structure of the protein persists even after passing the melting point. The reduction in bubble content resulted in improved scattering measurements with improved signal-to-noise ratios. This methodology is sensitive to detecting thermally induced conformational changes, even when the concentration and molecular weight of the stabilizer protein differ dramatically from the active pharmaceutical protein. Thus, it is a versatile technique for detecting both aggregation and conformational changes (refolding) of diluted proteins. It also contributes to studying the protein solubility [40]. The ability to determine melting points of dyes at extremely low concentrations without fluorescence allows for more accurate characterization of protein folding transitions in formulations where the active component is highly diluted, such as toxin-based therapeutics. It makes the technique suitable for quality control processes and formulation screening, providing novel insight into how stabilizers and excipients affect protein conformation. In addition, this approach may serve as a complementary method to support dye-based thermal shift assay.

## 5. Conclusions

This study demonstrates a dye-free RLS approach capable of detecting protein melting transitions from 1 pM to 1 uM concentrations. The method distinguishes conformational changes in BSA, HSA, BoNT-A and semaglutide peptide. Also, the method includes an unfolding kinetics well described by a single-exponential decay model without detecting aggregation at 1 pM. Operating beyond the sensitivity of conventional label-free techniques, RLS enables real-time monitoring of structural transitions at ultralow protein consentration. These findings establish RLS as a powerful and versatile tool for biopharmaceutical stability evaluation, formulation quality control, and studies correlating enzyme activity with conformational dynamics during thermal transition.

## Figures and Tables

**Table 1:** S/N ratio of BSA concentrations. Fluorescence emission at 20 °C (λ_exc_ = 295 nm).

[BSA]	S/N Ratio	Intensity at 350 nm
1 µM	1.37	411,192.7
1 nM	1.19	10,821.6
1 pM	1.02	5186.3

**Table 2:** Accuracy of the temperature set at the Peltier temperature controller.

Instruments	Temperature Range (°C)							
PT100	5	15	25	35	45	55	65	75
Peltier	5	15	24	33	43	52	62	72

**Table 3:** Melting points after correction.

Sample	HSA CSL	HSA	BoNT-A
Exp. melting point (°C)	52	48	65
Correction (°C )	54.05	49.89	67.57

## Data Availability

All raw data produced during this study are available at: Mendeley Data, V1, doi: 10.17632/975z7zg4jv.1.

## Supplementary Materials

The following supporting information can be downloaded at: https://www.mdpi.com/article/10.3390/biom15111579/s1, Figure S1: Resonance light scattering to detect the melting point at additional concentration. Figure S2: Melting points determination of incubated BOCOUTURE samples. Figure S3: Thermal transitions observed in Semaglutide (active component of Ozempic) 100-fold diluted. Table S1: Summary of Tm from BOCOUTURE samples at 20 °C and −20 °C.

## Abbreviations

The following abbreviations are used in this manuscript:

MP	Melting Point
BoNT-A	Botulinum Neuro Toxin A
BSA	Bovine Serum Albumin
HSA	Human Serum Albumin
RLS	Resonance Light Scattering
