# Supplementary material for: Observing Picomolar Protein Unfolding Using Resonance Light Scattering
Source: Biomolecules. 2025 Nov 10;15(11):1579. doi: 10.3390/biom15111579 (PMC12650451; doi:10.3390/biom15111579)
Supplement: Supplementary file 1 [file biomolecules-15-01579-s001.zip › biomolecules-3881533-supplementary.pdf]

Supporting Information

Observing picomolar protein unfolding using resonance light scattering

Alain Bolaño Alvarez [1,*], Kristian B. Arvesen [1], Kasper F. Hjuler [1], Peter Bjerring [1], and Steffen B. Petersen [1,*]

[1] Department of Dermatology and Venerology, Aalborg University Hospital. Hobrovej 18-20, Aalborg, 9000, Denmark.

* Correspondence: steffen1357@gmail.com, albolano88@gmail.com

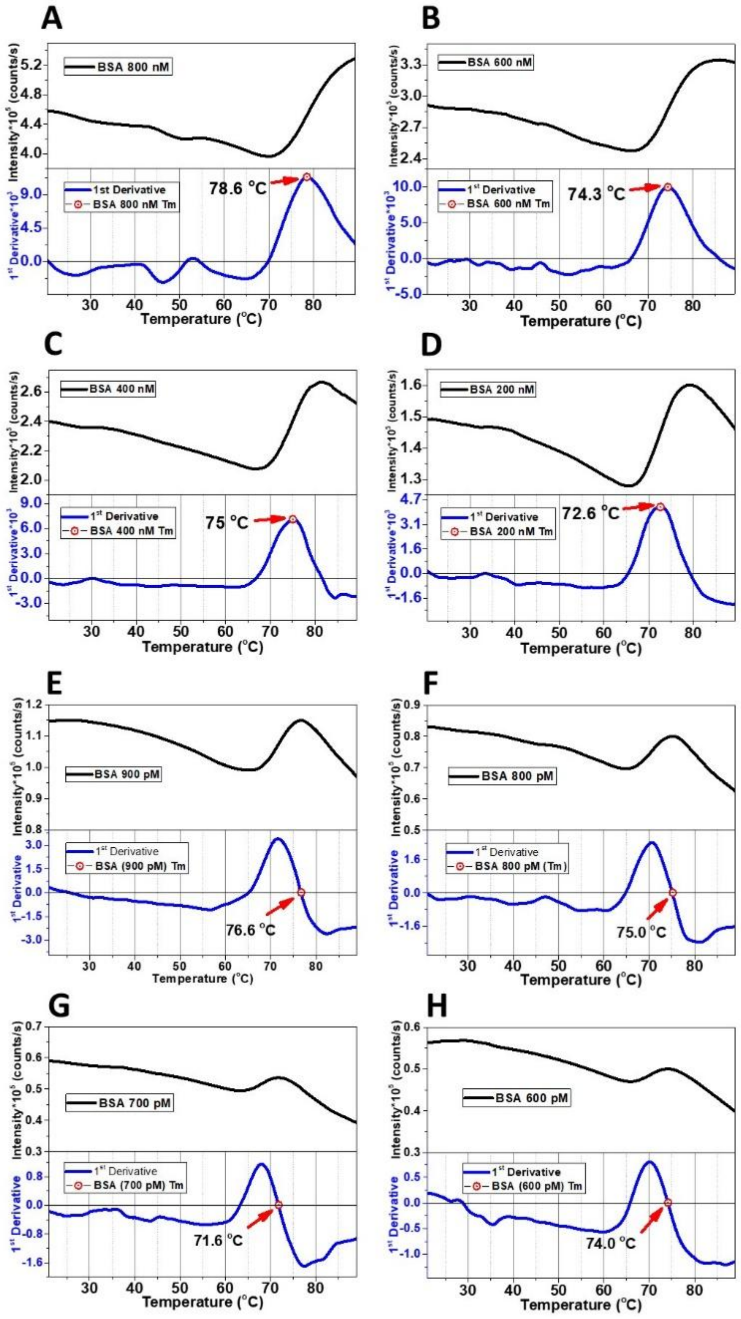

Figure S1: Resonance light scattering to detect the melting point at additional concentration. From 200 nM to 800 nM (**A-D**) and from 900 pM to 600 pM (**E-H**).

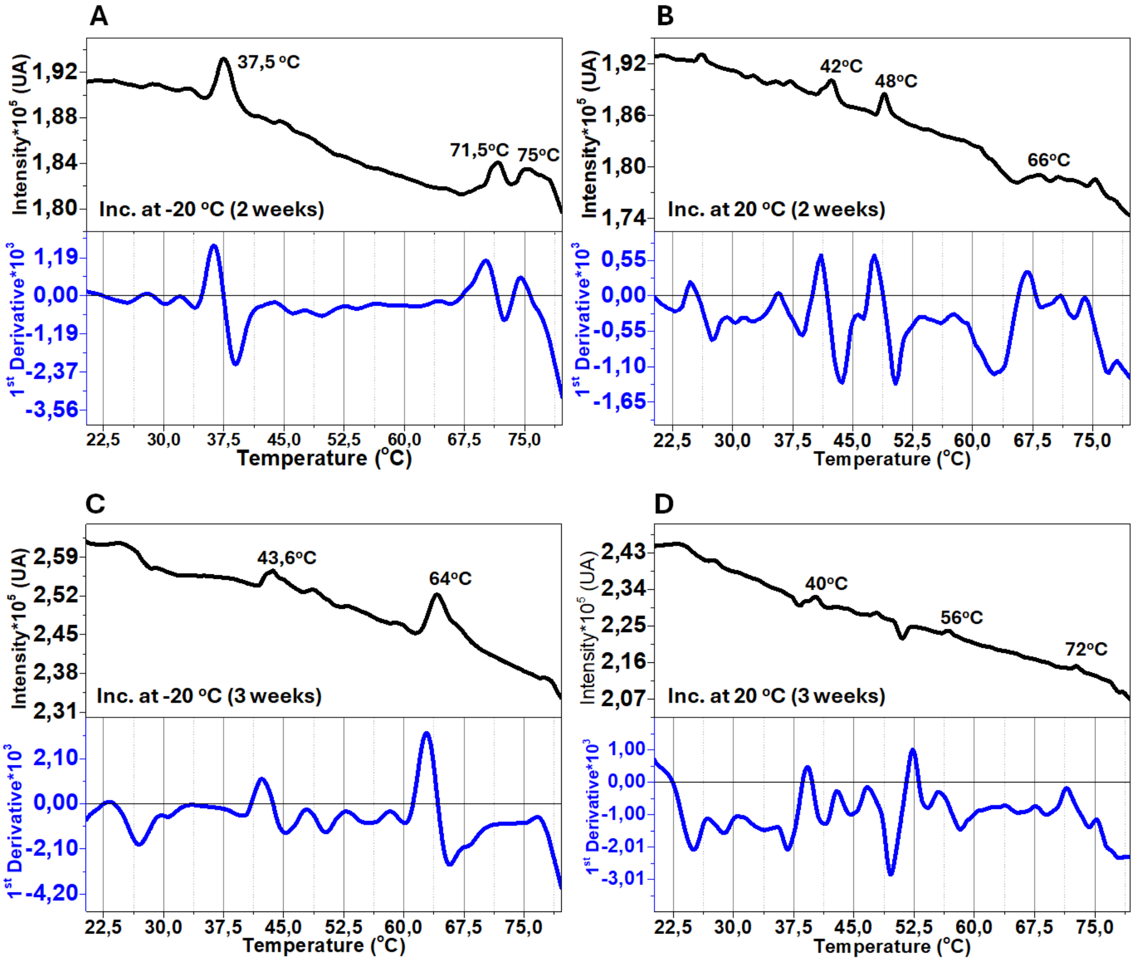

Figure S2: Melting points determination of incubated BOCOUTURE samples. Incubation for 2 weeks at -20 °C (**A**) and (**B**). Incubation during 3 weeks at -20 °C (**C**) and (**D**).

Table S1: Summary of Tm from BOCOUTURE samples at 20 °C and -20 °C.

Inc.	Inc. Temp. (°C)	Tm of BoTN-A	Tm of HSA	Remanent Tm
0 weeks	-----	*65*	*48*	-----
2 weeks	20	71	37.5	75
	-20	66.7	48	42 and 26
3 weeks	20	64	43.6	-----
	-20	72	40	56

Extension of the RLS Approach to Semaglutide (Ozempic API)

We have extended the RLS methodology to a peptide–lipid conjugate, semaglutide, the active component of Ozempic. Experiments were performed using a 100-fold diluted formulation, enabling detection of two distinct Tm. The first at ≈30 °C, corresponding to the soluble peptide fraction and consistent with the recommended storage temperature reported by Novo Nordisk, and a second, higher transition at ≈91 °C, which we attribute to an aggregated or "micellar-like" state unique to this lipidated peptide. We hypothesize that this high-temperature transition reflects the stabilization of a self-associated fatty acid core shielded by the GLP-1 peptide, resulting in enhanced structural stability upon heating. A separate manuscript currently under revision provides a detailed analysis of this system and further demonstrates the applicability of the RLS approach to this complex biopharmaceutical formulations.

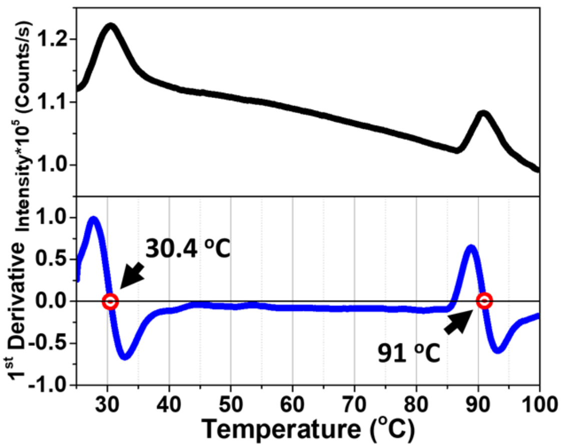

Figure S3: Thermal transitions observed in Semaglutide (active component of Ozempic) 100-fold diluted. Tm_1 = 30.4 °C and Tm_2 = 91 °C.
